# Low intensity vibration of ankle muscles improves balance in elderly persons at high risk of falling

**DOI:** 10.1371/journal.pone.0194720

**Published:** 2018-03-26

**Authors:** Nima Toosizadeh, Jane Mohler, Vladimir Marlinski

**Affiliations:** 1 Arizona Center on Aging, Department of Medicine, University of Arizona, Tucson, AZ, United States of America; 2 Division of Geriatrics, General Internal Medicine and Palliative Medicine, Department of Medicine, University of Arizona, Tucson, AZ, United States of America; 3 Department of Biomedical Engineering, University of Arizona, Tucson, Arizona, United States of America; 4 Division of Neurobiology, Barrow Neurological Institute, Phoenix, AZ, United States of America; University of L'Aquila, ITALY

## Abstract

In our study we examined postural performance of young healthy persons (HY), elderly healthy persons (HE), and elderly persons at high risk of falling (FR). Anterio-posterior (AP) and medio-lateral (ML) ankle and hip angular deviations, as well as linear displacements of the center of mass (COM) were assessed in persons standing with eyes either open or closed, while none, and 40 and 30 Hz vibrations were applied bilaterally to the ankle muscle gastrocnemius. During quiet standing with eyes open, balance parameters in FR group differed from those in healthy groups. ML ankle and hip angular deviations, as well as COM linear displacements were noticeably larger in FR group. During quiet standing with eyes closed, all balance parameters in participants of all groups had a clear trend to increase. During standing with eyes open, 40 Hz vibration increased all but one balance parameter within HY group, ankle angular deviations in HE group, but none in FR group. In response to 30 Hz vibration, only ankle angular deviations and COM linear displacements increased in HY group. There were no changes in both elderly groups. During standing with eyes closed, 40 and 30 Hz vibrations did not produce consistent changes in balance parameters in HY and HE groups. In FR persons, 40 Hz vibration did not change balance parameters. However, in FR groups, 30 Hz vibration decreased ankle and hip angular deviations, and COM linear displacements. The major result of the study is a finding that low intensity vibration of ankle muscles makes balance better in elderly persons at high risk of falling. This result is clinically relevant because it suggests that applying mild vibration to ankle muscles while standing and walking might benefit elderly persons, improving their postural performance and reducing a risk of unexpected falls.

## Introduction

One of serious problems challenging wellbeing of senior persons is a decline of their ability to flawlessly execute a complex skill for body equilibrium during standing and walking. The skill of maintaining equilibrium, which is learned and perfected through the first several years of human life, is based on a fine tuning of the activity of numerous components of the neuro-muscular system. With increasing age, efficiency of these components declines, and functioning of the system deteriorates. Muscles lose their mass, strength and power, which is known as sarcopenia and dynopenia [1,2]. Proprioceptors, muscle spindles and tendon organs, which detect muscle length and force, degenerate, and their number reduce [3,4]. The amount of neural fibers innervating muscles declines [5,6]. Velocity of signal transmission via surviving neural fibers reduces due to demyelinization [7,8]. The most critical processes related to aging, which affect motor performance, occur in the central nervous system. The brain shrinks, and reduces in volume with a rate of about 5 cm^3 a year [9,10,11,12,13,14].

The first manifestation of age-related deterioration of the skill for body equilibrium is an increase in body sway during standing [15,16,17]. As an increased magnitude of body movements raises instability of posture, elders can eventually lose balance and fall, suffering bone fractions, joint dislocations, concussions, and even death [18]. The magnitude of body sway depends on detection and processing of signals in somatosensory, visual and vestibular systems involved in maintenance of balance. It is suggested that deterioration of signal processing in these systems to some extent can be compensated by applying auxiliary random low-intensity stimulation, which produces the effect of so-called stochastic resonance [19,20,21]. Indeed, an application of this method to a division of the somatosensory system, which is responsible for cutaneous sensation, enhances postural stability during both standing and walking. Sole vibrations with random frequencies and magnitude below a threshold for producing perception of the activation of plantar mechanoreceptors improved balance and gait in healthy elderly people [22,23]. Increased sway of the body in patients with diabetic neuropathy and stroke was reduced using subsensory mechanical noise applied to soles of feet [24]. Stimulation with a low level electrical noise applied at the knee, which presumably activated cutaneous receptors, was found to enhance balance performance in healthy elderly persons [25]. At the same time, consequences of low intensity stimulation on another division of the somatosensory system, which is responsible for sensation of muscle and tendon length and force, are not well understood yet. Potential of such stimulation for improvements in postural stability in elders still has to be verified.

An excitation of sensory structures (muscle spindles) detecting changes in muscle length can be achieved using vibratory stimulation of bellies or tendons of muscles. It is known that vibratory stimulation of ankle muscles, which are of the foremost importance for maintenance of balance during biped standing in humans, elicit sway of the body [26,27]. The magnitude of sway depends on the frequency of vibration. In healthy persons, the maximal postural effect is produced by 80–100 Hz vibrations; it diminishes with a decrease in frequency, and is not visible with vibrations with frequencies below 30–40 Hz [26,28]. Noteworthy, motor responses to near-threshold vibratory stimulation may not be consistent and vary between persons [29,30]. It was reported, for example, that vibration of the muscle triceps surae with frequencies below 20 Hz produced body sway in the direction opposite to sway elicited by high frequency vibration in 20% of young healthy adults participated in experiments [31].

The goal of our study was to elucidate whether low intensity stimulation of proprioceptors of antigravitaional muscles can improve postural parameters in persons, whose neuro-muscular system is prone to malfunctions due to aging. While achieving this goal we had to clarify whether effects of low intensity proprioceptive stimulation vary depending on age and health of persons. For this purpose we compared postural parameters during standing between healthy young persons, whose neuro-muscular system presumably functions flawlessly, and healthy elderly persons, whose neuro-muscular system might experience minor age-related changes, which however did not compromise maintenance of balance. We also compared postural parameters between both groups of healthy persons and older-elderly persons, whose neuro-muscular system underwent substantial age-related changes, resulted in high risk of falling. To examine postural effects of proprioceptive stimulation we applied bilaterally low frequency vibration to the ankle extensor, gastrocnemius muscle.

We hypothesized that occurrence of postural responses and their quality would vary between participants depending on age and health status. We expected that in healthy young persons, even low intensity vibration applied to muscles would disturb function of well-balanced neuro-muscular system, and produce body sway expanding the magnitude of balance parameters during standing. At the same time, in healthy elderly persons, low intensity muscle vibration might be ineffective to cause distinct changes in balance parameters during standing. We hypothesized that in older-elderly persons at high risk of falling, low intensity muscle vibration might have the effect opposite to that in young healthy persons. Such muscle vibration might be insignificant to produce motor responses increasing sway of the body during standing. However, afferent signals elicited by this vibration can be sufficient enough to elevate a subthreshold activity of motor neurons of the parent muscle, and facilitate responses of these neurons to afferent signals due to natural postural perturbations. As a result, low intensity muscle vibration in fall risk elderly persons could be followed by improvements in balance during standing. We suggested that such effect of low intensity proprioceptive stimulation can benefit maintenance of equilibrium in older-elderly persons, whose deteriorated neuro-muscular system became prone to malfunctions resulting in falls.

## Materials and methods

### Participants

Thirty subjects, divided into three groups, participated in the study. One group consisted of ten healthy young persons (HY), five males and five females (age 23.3±2.3 years; stature 173±10 cm; body mass 70.8±16.7 kg). Second group consisted of ten healthy elderly persons (HE), three males and seven females (age 72.9±2.8 years; stature 165±11 cm; body mass 64.7±8.4 kg), who did not report falls in the past. Third group consisted of ten older-elderly persons at a high risk of falling (FR, 3±4.6 falls within one year), three males and seven females (age 83.6±9.6 years; stature 166±11 cm; body mass 65.2±16.4 kg). Persons, who were included in HY and HE groups did not answer affirmatively to any questions, while persons included in FR group gave affirmative answers to two or more of the questions of the Center for Disease Control and Prevention’s STEADI Risk for Falling Assessment [32]: (i) Have you fallen in the past year? (ii) Are you worried about falling? (iii) Do you feel unsteady when you are walking? (iv) Have you had two or more falls? Potential participants were excluded if they had a history of disorders associated with severe motor deficits and balance performance, stroke, Parkinson’s disease, diabetic neuropathy, vestibular diseases, and severe arthritis in lower-extremities.

### Ethic statement

The experimental protocol was approved by the University of Arizona’s Review Boards in accordance with the principles of the 1964 Helsinki Declaration. All participants gave their informed consent to participate in experiments.

### Procedures

**Fig 1:**
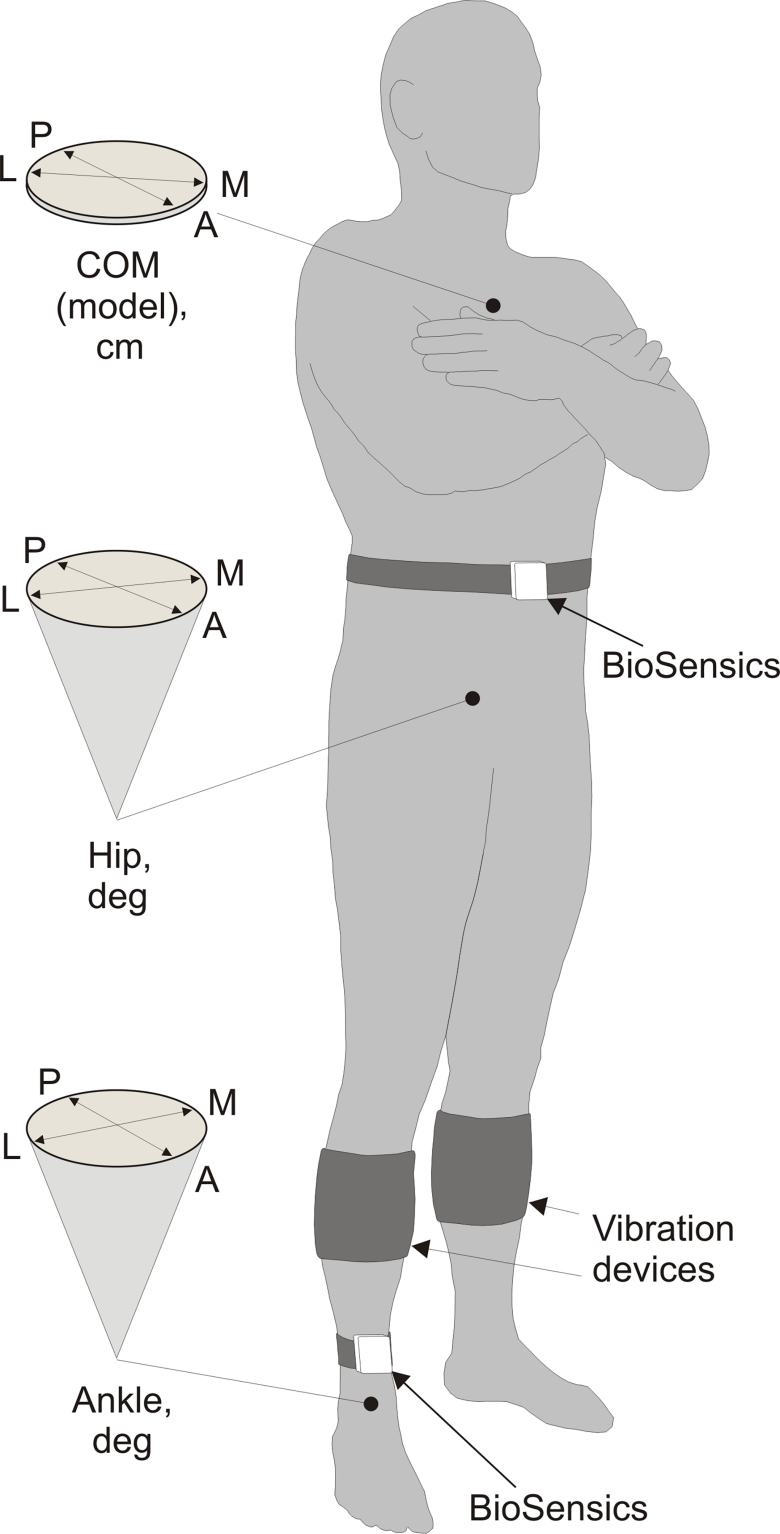
Experimental design. During an experiment, gyroscope sensors (BioSensics) were positioned on person’s right shin and waist. Sensors measured ankle and hip angular deviations in two orthogonal directions. The angular data were integrated into the biomechanical model of the human body for estimating linear displacements of the center of mass of the body (COM). Diagrams on the left depict angular and linear balance parameters recorded. Vibration devices were placed on both calves of a person. The vibrators were positioned above bellies of gastrocnemius muscles. Indices: A–anterior, P–posterior, L–lateral, M–medial.

During experiments participants stood upright on a firm surface. Participants were asked to keep arms folded and position their feet as close together as possible, but without touching each other (Fig 1) [33,34]. In one part of experiments, participants stood with eyes open without gaze fixation on a specific target, and in another part, participants stood with eyes closed.

In each participant, balance parameters were assessed in eight 30 seconds trials. Initial two trials, one with eyes open and another with eyes closed, were completed before placing vibratory device on participant’s calves to obtain reference balance parameters of a subject. After placing vibratory devices, participants were exposed to one-minute vibration of their ankle muscles to get used to the novel mechanical stimulus prior to vibrational balance tests. Then, two trials were completed, in one of which participants stood with eyes open and in another with eyes closed, but no vibration was applied. These two trials were used to assess subject’s baseline balance parameters during experiments. In next two trials, 30 Hz muscle vibration was applied when a participant stood with eyes open and when a participant stood with eyes closed. In final two trials, 40 Hz muscle vibration was applied when a participant stood with eyes open and when a participant stood with eyes closed. Between trials that involved vibration, participants had a two-minute rest period in a sitting rest position to minimize the residual effects of vibration on subsequent balance behaviors [35].

### Vibratory stimulation

Mechanical stimulation of proprioceptors of gastrocnemius muscles was produced by vibration devices placed on right and left calves (Fig 1). The device contained a vibrator, which was a small direct drive motor with an attached eccentric load (total mass 33 g), enclosed into a plastic barrel (30 mm height, 40 mm width, and 50 mm length). The motor was powered with two 1.5 V AAA batteries. The vibrator and batteries were enclosed in a sleeve, 13 cm in width, made of soft fabric with an attached elastic band. To enable power and select vibration frequency, a manual switch was attached to the sleeve. The total mass of the vibration device was 140 g. During experiments, each device was placed on a calf in a way that positioned a vibrator above the belli of gastrocnemius muscle. Effects of mechanical proprioceptive stimulation were tested with two dominant frequencies of vibration, 30 and 40 Hz. As discussed above in the Introduction, the vibration frequency of 30 Hz is near threshold, and 40 Hz is the frequency just above threshold for eliciting postural response in young healthy subjects standing upright. The amplitude of vibrator’s movements was about 1 mm.

### Recordings

A wearable sensor system for balance assessment (BalanSens TM; BioSensics LLC, Boston, MA) was used to evaluate postural parameters of participants during experiments. Details of recording technique was described in details in our previous publications [33,34]. Ankle and hip angular deviations in anterio-posterior (AP) and medio-lateral (ML) directions were measured with two triaxial gyroscope sensors (Fig 1). The sensors were attached with elastic Velcro straps to person’s right shin and waist, in locations identical across all participants (Fig 1). The joint’s angular data, as well as anthropometric data of a participant (stature and body mass), were integrated into the biomechanical model of the human body for estimating AP and ML linear displacements of the center of mass of the body (COM) [36]. In addition to angular and linear parameters, BalanSense software estimated and reported ankle and hip angular sway (deg^2), as well as a linear COM sway (cm^2) [36].

### Statistical data analysis

Baseline balance parameters in three groups of participants standing quietly with eyes open or eyes closed were compared using ANOVA. Differences in parameters between groups were validated using post-hoc Tukey HSD test. In each group of participants, balance parameters assessed during standing with both eyes open and eyes closed were compared with repeated measures MANOVA. Effects of muscle vibration on balance parameters while standing with both eyes open and eyes closed were assessed in each group of participants. In each individual group, balance parameters during quiet standing and corresponding parameters during 30 and 40 Hz vibrations were compared using repeated measures MANOVA. In addition, parametric statistical analysis of effects of muscle vibration was complemented with non-parametric χ^2^ test of changes in balance parameters. For this purpose we counted the number of participants of each group, whose balance parameter increased or decreased during muscle vibration as compared to parameter during quiet standing. For all procedures of statistical comparison of the data, a significance level was set to p<0.05. Balance parameters in each group of participants are shown in the text as an average ± standard deviation. Data analysis was completed using EXCEL (Microsoft, Redmont, WA) and JMP (Version 11, SAS Institute Inc., Cary, NC),

## Results

### Balance parameters during standing with and without vibration devices on calves before application of vibratory stimulation

Vibratory devices placed on calves were enclosed in soft sleeves, which gently pressured the skin and apparently stimulated cutaneous mechanoreceptors. Some of these receptors adapt rapidly, while other adapt slowly to mechanical stimuli of constant intensity. We found necessary to clarify whether mild constant pressure of calf’s skin affected balance parameters in our experiments. To this end, we compared balance parameters in HY, HE, and FR participants when they did or did not wear sleeves while standing with eyes open and eyes closed. We found only marginal differences between parameters measured when participants did and did not wear sleeves. Small, though statistically significant differences were found in only two parameters in the HE group. In this group, angular ML ankle deviations and linear ML COM displacements were larger when participants wore sleeves during standing with eyes closed. There were no differences between parameters in other two groups. Based on this comparative analysis, we concluded that sleeves positioned on calves did not substantially affect postural performance of participants in our study. Accordingly, we considered the data obtained during quiet standing with vibratory devices positioned on calves prior to an application of vibrations as adequate baseline balance parameters for assessment of effects of ankle muscle vibration on posture.

### Balance parameters during quiet standing with eyes open and eyes closed

**Fig 2:**
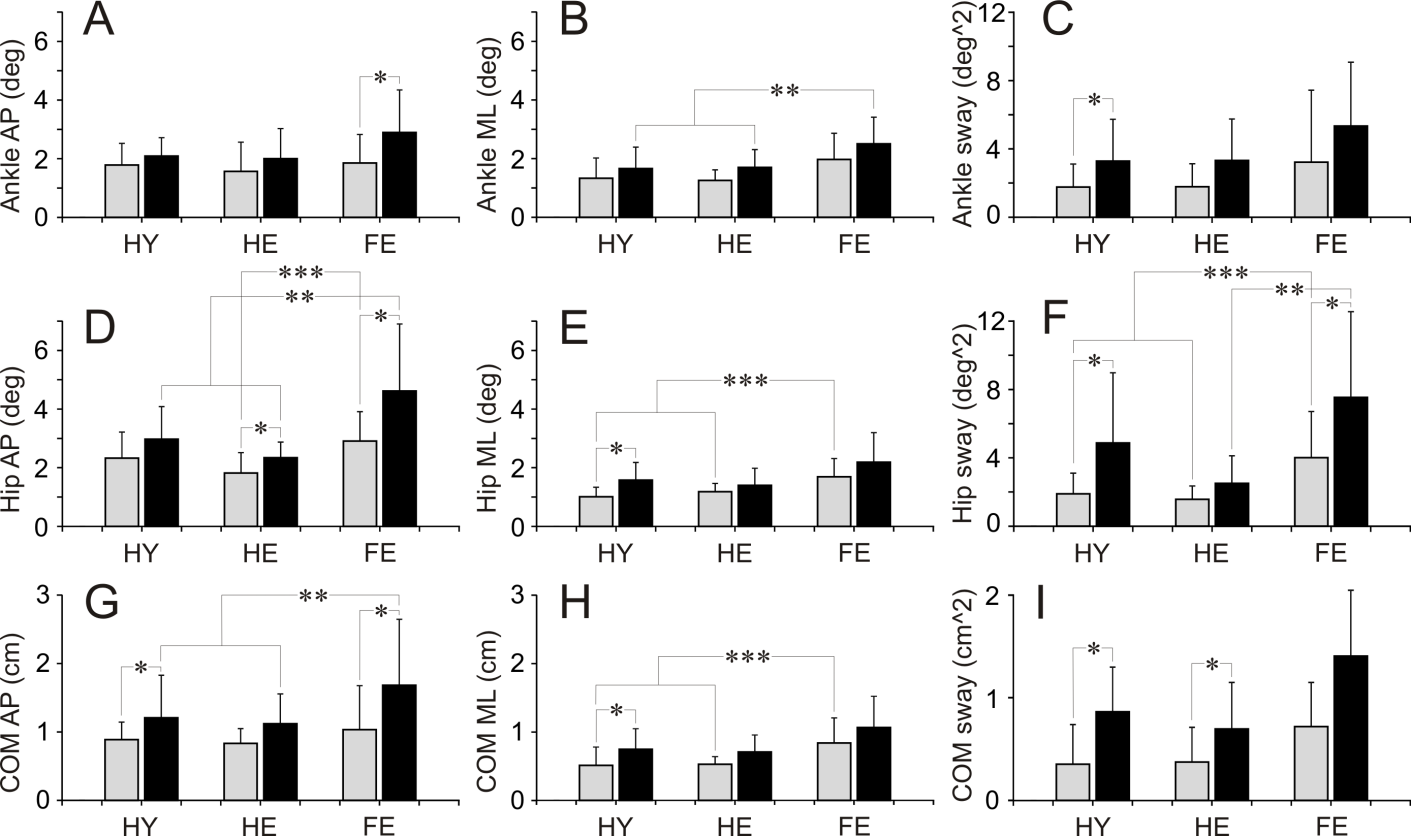
Balance parameters during quiet standing with eyes open and eyes closed. **(A**, **B**, **C)** Ankle anterio-posterior (AP), medio-lateral (ML) deviations (deg), and sway (deg^2), respectively. **(D**, **E**, **F)** Hip anterio-posterior (AP), medio-lateral (ML) deviations (deg), and sway (deg^2), respectively. **(G**, **H**, **I)** COM anterio-posterior (AP), medio-lateral (ML) displacements (cm), and sway (cm^2), respectively. Grey bars depict parameters measured during standing with eyes open. Black bars depict parameters measured during standing with eyes closed. A star (*) indicates parameters in a group, which were significantly different during standing with eyes open and eyes closed (repeated measures MANOVA). Two stars (**) indicate parameters of different groups, which were significantly different during standing with eyes closed (Tukey HSD test). Three stars (***) indicate parameters of different groups, which were significantly different during standing with eyes open (Tuckey HSD test). HY–healthy young persons. HE–healthy elderly persons. FR–elderly persons at high risk of falling persons.

Baseline balance parameters of participants during quiet standing are shown in Table 1 and Fig 2. Statistical differences in parameters between three groups of participants, and differences in every single parameter within each group during standing with eyes open or eyes closed are presented in Table 1. Statistically significant differences between parameters validated with Tukey HSD test are indicated in Fig 2.

**Table 1:** Baseline balance parameters during standing with eyes open and eyes closed. Difference in a parameter between groups assessed using ANOVA. Difference in a parameter within a group during standing with eyes open and eyes closed assessed using repeated measures MANOVA. Underlined bold characters highlight statistically significant difference between the balance parameter in two conditions (p<0.05). Bold characters highlight difference between the balance parameter that was just above statistical significance (0.1<p<0.05).

	Healthy Young	Healthy Elderly	Fall Risk	Group difference
**Ankle AP (deg)**				
Eyes open	1.78±0.74	1.57±1.0	1.85±0.97	F(2,9) = 0.27 p = 0.7622
Eyes closed	2.09±0.63	2.0±1.02	2.90±1.45	F(2,9) = 2.07 p = 0.1457
	F(1,9) = 2.92 p = 0.1214	F(1,9) = 2.92 p = 0.2609	F(1,9) = 5.93 **p =** **0.0377**	
**Ankle ML (deg)**				
Eyes open	1.33±0.69	1.26±0.36	1.97±0.89	F(2,9) = 2.95 **p = 0.0692**
Eyes closed	1.66±0.73	1.70±0.61	2.51±0.91	F(2,9) = 3.99 **p =** **0.0304**
	F(1,9) = 0.97 p = 0.3498	F(1,9) = 4.58 **p = 0.0610**	F(1,9) = 2.76 p = 0.1318	
**Ankle sway (deg^2)**				
Eyes open	1.76±1.35	1.79±1.35	3.23±4.21	F(2,9) = 0.99 p = 0.3854
Eyes closed	3.29±2.44	3.32±2.43	5.34±3.73	F(2,9) = 1.61 p = 0.2184
	F(1,9) = 7.24 **p =** **0.0248**	F(1,9) = 4.42 **p = 0.0650**	F(1,9) = 1.80 p = 0.2126	
**Hip AP (deg)**				
Eyes open	2.33±0.89	1.82±0.70	2.91±1.0	F(2,9) = 3.89 **p =** **0.0327**
Eyes closed	2.97±1.11	2.34±0.53	4.62±2.28	F(2,9) = 6.14 **p =** **0.0063**
	F(1,9) = 4.76 **p = 0.0571**	F(1,9) = 7.63 **p =** **0.0220**	F(1,9) = 5.14 **p =** **0.0495**	
**Hip ML (deg)**				
Eyes open	1.01±0.32	1.18±0.28	1.69±0.62	F(2,9) = 6.59 **p =** **0.0047**
Eyes closed	1.58±0.60	1.40±0.58	2.19±1.01	F(2,9) = 2.97 **p = 0.0683**
	F(1,9) = 12.50 **p =** **0.0064**	F(1,9) = 1.32 p = 0.2805	F(1,9) = 2.56 p = 0.1444	
**Hip sway (deg^2)**				
Eyes open	1.90±1.21	1.58±0.78	4.01±2.70	F(2,9) = 5.62 **p =** **0.0091**
Eyes closed	4.87±4.11	2.51±1.61	7.54±5.01	F(2,9) = 4.26 **p =** **0.0246**
	F(1,9) = 6.51 **p =** **0.0311**	F(1,9) = 5.12 **p = 0.0500**	F(1,9) = 5.32 **p =** **0.0465**	
**COM AP (cm)**				
Eyes open	0.89±0.39	0.83±0.34	1.03±0.43	F(2,9) = 0.74 p = 0.4850
Eyes closed	1.21±0.43	1.12±0.45	1.68±0.64	F(2,9) = 3.43 **p =** **0.0472**
	F(1,9) = 8.46 **p =** **0.0174**	F(1,9) = 3.67 **p = 0.0876**	F(1,9) = 8.29 **p =** **0.0182**	
**COM ML (cm)**				
Eyes open	0.50±0.26	0.53±0.11	0.82±0.36	F(2,9) = 4.63 **p =** **0.0187**
Eyes closed	0.73±0.30	0.69±0.24	1.04±0.45	F(2,9) = 3.24 **p = 0.0550**
	F(1,9) = 9.10 **p =** **0.0145**	F(1,9) = 4.30 **p = 0.0679**	F(1,9) = 2.00 p = 0.1905	
**COM sway (cm^2)**				
Eyes open	0.35±0.26	0.37±0.22	0.72±0.64	F(2,9) = 2.40 p = 0.1098
Eyes closed	0.86±0.62	0.69±0.43	1.40±0.96	F(2,9) = 2.74 **p = 0.0822**
	F(1,9) = 10.98 **p =** **0.0090**	F(1,9) = 5.27 **p =** **0.0473**	F(1,9) = 3.69 **p = 0.0870**	

Baseline balance parameters during standing with eyes open were almost identical in two groups of healthy participants, HY and HE. In contrast, a range of several balance parameters in FR group exceeded those of HY and HE groups. The difference was most apparent in hip movements. In FR group, hip AP and ML angular deviations as well as sway were significantly larger than those in both HY and HE groups (Table 1. Fig 2D, 2E and 2F). In addition, ankle ML angular deviations in FR group exceeded almost significantly those in HY and HE groups (Table 1. Fig 2B). Finally, in FR group, COM ML linear displacements were significantly larger in comparison to HY and HE groups (Table 1. Fig 2H).

When participants stood with eyes closed, a range of all balance parameters showed a trend to increase in all groups. In HY group, ankle sway, hip ML angular deviations and sway, as well as all COM linear displacements and sway increased significantly (Table 1. Fig 2C, 2E, 2F, 2G, 2H and 2I). In addition, an increase in hip AP angular deviations almost reached a level of statistical significance (Table 2). In HE group, an increase in hip AP angular deviation and COM sway were statistically significant, while ankle ML angular deviations and sway, hip sway, and COM linear displacements were just above a level of significance (Table 1. Fig 2D and 2I). In FR group, a statistically significant increase was found in ankle AP angular deviations, hip AP angular deviations and sway, and COM AP linear displacements (Table 1. Fig 2A, 2D, 2F and 2G). Besides, an increase in COM sway was close to the level of significance (Table 1. Fig 2I). In all three groups of participants, ranges of ankle and hip angular sway as well as COM linear sway expanded substantially, reaching approximately a two-fold increase.

**Table 2:** Ankle angular deviations and sway during standing without and with calves’ vibration. Difference in a parameter before (baseline) and during vibration assessed using repeated measures MANOVA. Differences in frequencies of parameter increases and decreases that occurred during vibration assessed using χ^2^ test. Underlined bold characters highlight statistically significant difference between the balance parameter in two conditions (p<0.05). Bold characters highlight difference between the balance parameter that was just above statistical significance (0.1<p<0.05).

			Healthy Young	Healthy Elderly	Fall Risk
**Ankle AP** **(cm)**	Eyes open	Baseline	1.78±0.74	1.57±1.0	1.85±0.97
		40 Hz	3.30±1.52	2.23±0.61	2.13±1.0
			F(1,9) = 8.37 **p =** **0.0178**	F(1,9) = 8.84 **p =** **0.0156**	F(1,9) = 0.43 p = 0.5271
			*(+) 8 (-) 2* ***p = 0*.*056***	*(+) 8 (-) 2* ***p = 0*.*0568***	*(+) 6 (-) 4 p = 0*.*5271*
		30 Hz	2.68±1.45	2.26±0.67	2.49±1.62
			F(1,9) = 3.14 p = 0.1101	F(1,9) = 4.51 **p = 0.0628**	F(1,9) = 3.29 p = 0.1030
			*(+) 6 (-) 4 p = 0*.*5271*	*(+) 8 (-) 2* ***p = 0*.*0568***	*(+) 5 (-) 5 p = 1*.*0*
	Eyes closed	Baseline	2.09±0.63	1.02±2.09	2.90±1.45
		40 Hz	3.22±1.38)	3.22±1.38)	2.56±1.24
			F(1,9) = 5.00 **p = 0.0522**	F(1,9) = 1.49 p = 0.2529	F(1,9) = 1.62 p = 0.2355
			*(+) 8 (-) 2* ***p = 0*.*0568***	*(+) 8 (-) 2* ***p = 0*.*0568***	*(+) 2 (-) 8* ***p = 0*.*0568***
		30 Hz	2.91±1.69	2.15±0.49	2.23±0.72
			F(1,9) = 2.2990	F(1,9) = 0.2474	F(1,9) = 5.29
			p = 0.1638	p = 0.6308	**p =** **0.0471**
			*(+) 6 (-) 4 p = 0*.*5271*	*(+) 6 (-) 4 p = 0*.*5271*	*(+) 2 (-) 8* ***p = 0*.*0568***
**Ankle ML** **(cm)**	Eyes open	Baseline	1.33±0.69	1.26±0.36	1.97±0.89
		40 Hz	2.39±0.83	2.14±0.82	1.99±0.64
			F(1,9) = 10.22 **p =** **0.0109**	F(1,9) = 9.00 **p =** **0.0149**	F(1,9) = 0.01 p = 0.9565
			*(+) 9 (-) 1* ***p =*** **0.0114**	*(+) 8 (-) 2* ***p = 0*.*0568***	*(+) 5 (-) 5 p = 1*.*0*
		30 Hz	2.56±0.92	1.60±0.45	2.0±1.0
			F(1,9) = 16.21 **p =** **0.0030**	F(1,9) = 4.01 **p = 0.0763**	F(1,9) = 0.01 p = 0.9213
			*(+) 9 (-) 1* ***p =*** ***0*.*0114***	*(+) 7 (-) 3 p = 0*.*2059*	*(+) 4 (-) 6 p = 0*.*5271*
	Eyes closed	Baseline	1.66±0.73	1.70±0.61	2.51±0.91
		40 Hz	2.32±1.11	2.23±0.39	2.38±0.49
			F(1,9) = 2.19 p = 0.1734	F(1,9) = 5.01 **p = 0.0519**	F(1,9) = 0.31 p = 0.5910
			*(+) 8 (-) 2* ***p = 0*.*0568***	*(+) 8 (-) 2* ***p = 0*.*0568***	*(+) 5 (-) 5 p = 1*.*0*
		30 Hz	2.31±0.52	2.04±0.80	2.23±0.60
			F(1,9) = 5.7602 **p = 0.0399**	F(1,9) = 0.95 p = 0.3545	F(1,9) = 1.93 p = 0.1984
			*(+) 9 (-) 1* ***p =*** ***0*.*0114***	*(+) 6 (-) 4 p = 0*.*5271*	*(+) 2 (-) 8* ***p = 0*.*0568***
**Ankle sway** **(cm^2)**	Eyes open	Baseline	1.76±1.35	1.79±1.35	3.23±4.21
		40 Hz	5.81±3.83	3.06±2.02	2.89±1.89
			F(1,9) = 16.16 **p =** **0.0030**	F(1,9) = 3.29 p = 0.1031	F(1,9) = 0.06 p = 0.8158
			*(+) 8 (-) 2* ***p = 0*.*0568***	*(+) 8 (-) 2* ***p = 0*.*0568***	*(+) 5 (-) 5 p = 1*.*0*
		30 Hz	5.85±4.86	3.02±1.52	3.25±3.16
			F(1,9) = 6.3004 **p =** **0.0333**	F(1,9) = 4.05 **p = 0.0751**	F(1,9) = 0.01 p = 0.9809
			*(+) 7 (-) 3 p = 0*.*2059*	*(+) 8 (-) 2* ***p = 0*.*0568***	*(+) 4 (-) 6 p = 0*.*5271*
	Eyes closed	Baseline	3.29±2.44	3.32±2.43	5.34±3.73
		40 Hz	6.70±5.51	3.85±1.80	4.63±3.88
			F(1,9) = 2.94 p = 0.1204	F(1,9) = 0.22 p = 0.6514	F(1,9) = 1.53 p = 0.2471
			*(+) 8 (-) 2* ***p = 0*.*0568***	*(+) 6 (-) 4 p = 0*.*5271*	*(+) 3 (-) 7 p = 0*.*2059*
		30 Hz	6.82±6.61	3.03±1.77	3.52±1.62
			F(1,9) = 2.79 p = 0.1292	F(1,9) = 0.08 p = 0.7807	F(1,9) = 4.49 **p = 0.0632**
			*(+) 7 (-) 3 p = 0*.*2059*	*(+) 4 (-) 6 p = 0*.*5271*	*(+) 3 (-) 7 p = 0*.*2059*

During standing with eyes closed, there were no differences between balance parameters in two groups of healthy participants. In contrast to these two groups, participants of FR group had a significantly higher range of ankle ML angular deviations, hip AP angular deviations and sway, as well as COM AP linear displacements (Table 1. Fig 2B, 2D, 2F and 2G). In addition, in FR group, ML hip angular deviations and COM linear displacements, as well as COM sway almost significantly exceeded these parameters in HY and HE (Table 1. Fig 2E, 2H and 2I).

### Vibration applied to ankle muscles. Balance parameters during standing with eyes open

**Fig 3:**
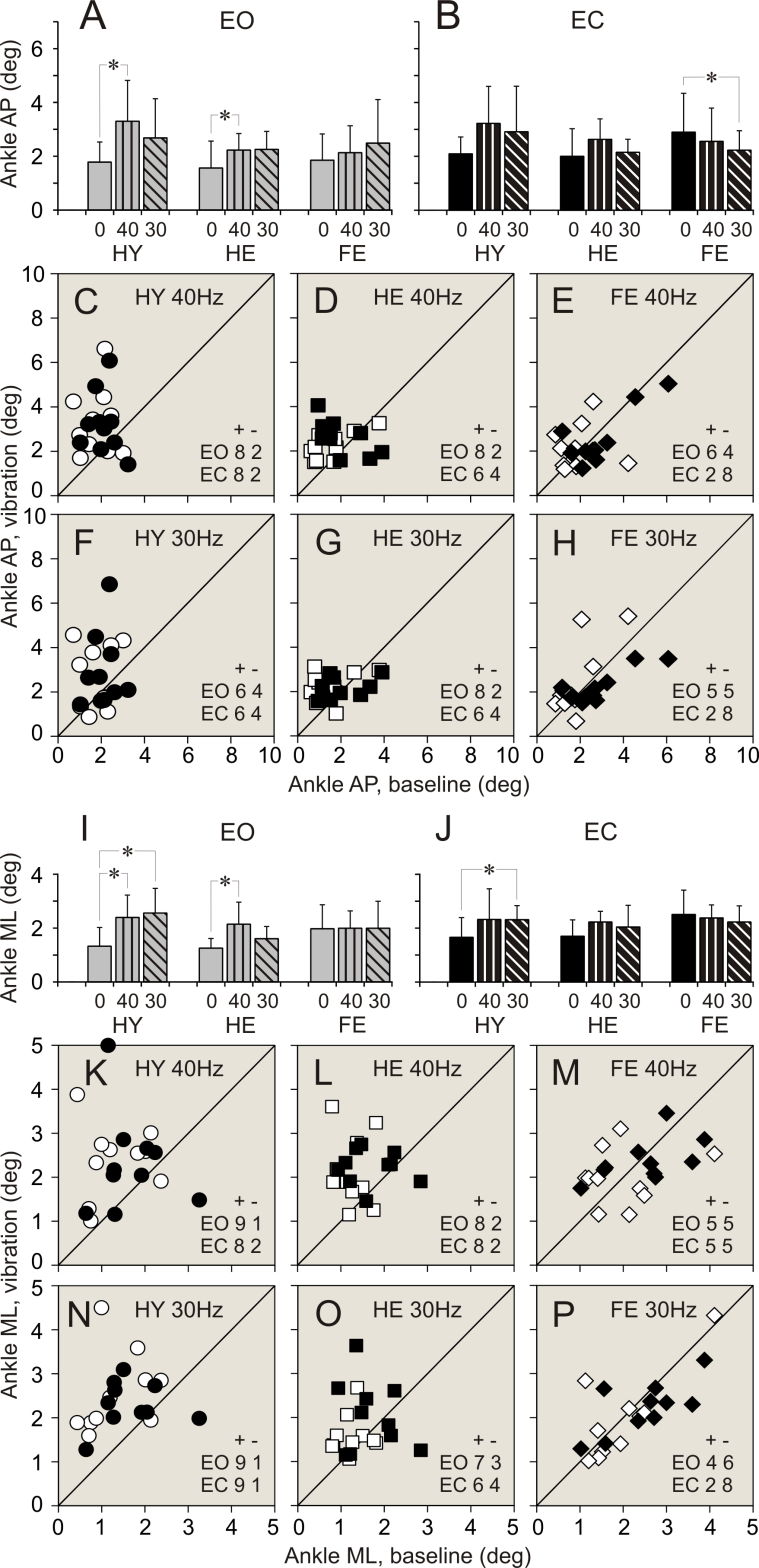
Ankle angular deviations during standing with and without vibration of ankle muscles. **(A**, **B)** Ankle anterio-posterior (AP) deviations during standing with eyes open (EO), and eyes closed (EC), respectively. Bars depict average of a parameter in a group. Vertical line attached to a bar indicates standard deviation of a parameter. Grey bars–parameters during quiet standing, bars with vertical stripes–parameters during 40 Hz vibration, bars with diagonal stripes–parameters during 30 Hz vibration. Vibration frequencies are indicated below bars. HY–healthy young persons. HE–healthy elderly persons. FR–older-elderly persons at high risk of falling persons. A star (*) indicate the parameter in a group which was significantly different during quiet standing and during standing with applied vibration (repeated measures MANOVA). **(C**, **D**, **E)** Individual AP deviations during standing with 40 Hz vibration plotted against deviations during quiet standing in participants of HY, HE, and FR groups, respectively. White symbols depict data collected during standing with eyes open. Black symbols depict data collected during standing with eyes closed. Circles represent data in HY group. Squares represent data in HE group. Diamonds represent data in FR group. Numbers in the right low corner of each plot indicate quantity of members of a group whose parameter increased (+) or decreased (-) during calves’ vibration while standing with eyes open (EO) or eyes closed (EC). A diagonal line is a reference line of unity. **(F**, **G**, **H)** Individual AP deviations measured during standing with 30 Hz vibration plotted against deviations during quiet standing in participants of HY, HE, and FR groups, respectively. Designations are similar to those in plots above. **I**, **J)** Ankle medio-lateral (ML) deviations during standing with eyes open (EO), and eyes closed (EC), respectively. Designations as in panels A and B. **(K**, **L**, **M)** Individual ML deviations measured during standing with 40 Hz vibration plotted against deviations during quiet standing in participants of HY, HE, and FR groups, respectively. Designations as in panels C, D and E. **(N**, **O**, **P)** Individual ML deviations measured during standing with 30 Hz vibration plotted against deviations during quiet standing in participants of HY, HE, and FR groups, respectively. Designations as in panels C, D and E.

**Fig 4:**
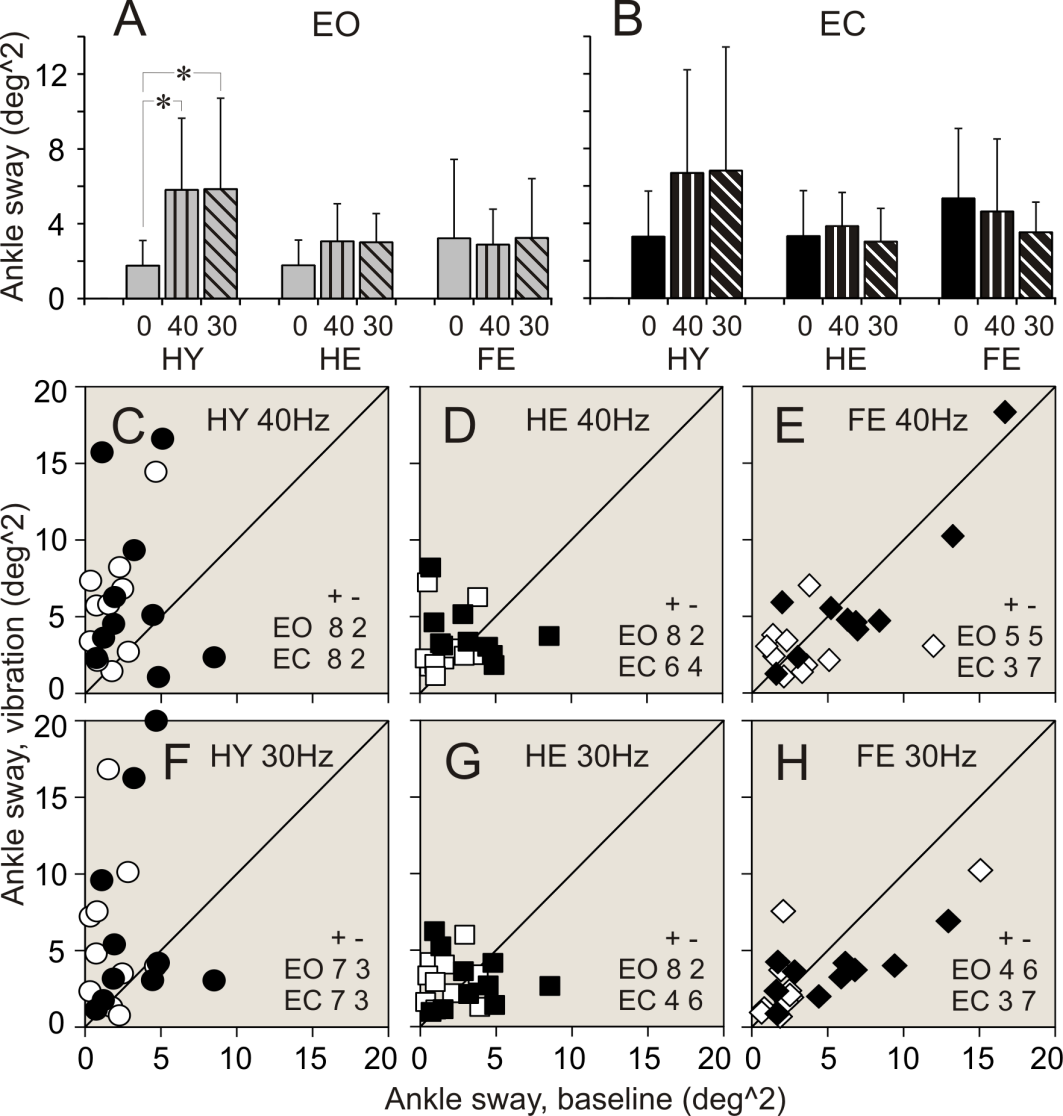
Ankle angular sway during standing with and without vibration of ankle muscles. **(A**, **B)** Ankle sway during standing with eyes open (EO), and eyes closed (EC), respectively. **(C**, **D**, **E)** Individual ankle sway during standing with 40 Hz vibration plotted against sway during quiet standing in participants of HY, HE, and FR groups, respectively. **F**, **G**, **H**: Individual ankle sway measured during standing with 30 Hz vibration plotted against sway during quiet standing in participants of HY, HE, and FR groups, respectively. Designations as in Fig 3.

**Fig 5:**
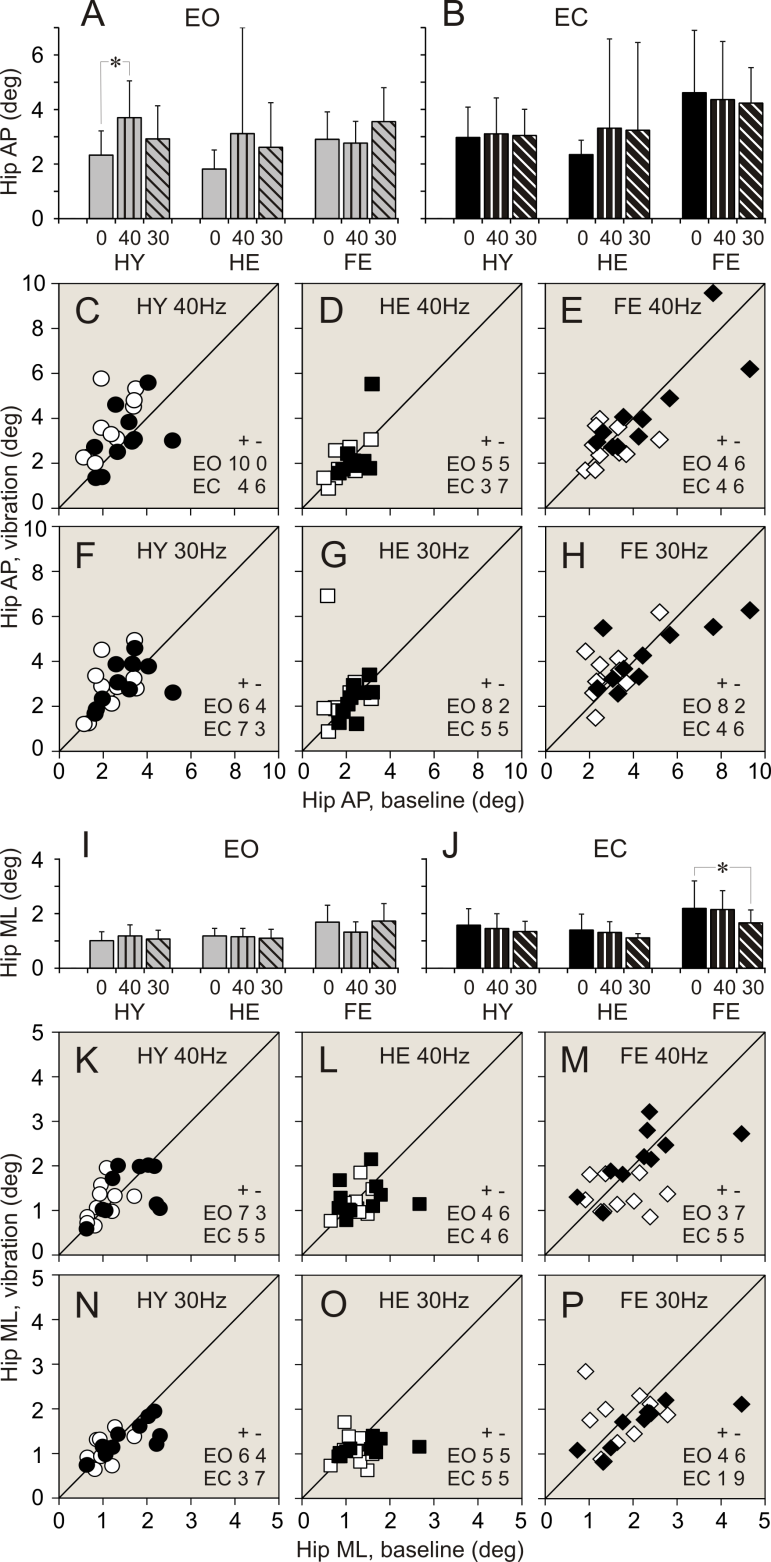
Hip angular deviations during standing with and without vibration of ankle muscles. **(A**, **B)** Hip anterio-posterior (AP) deviations during standing with eyes open (EO), and eyes closed (EC), respectively. **(C**, **D**, **E)** Individual AP deviations during standing with 40 Hz vibration plotted against deviations during quiet standing in participants of HY, HE, and FR groups, respectively. **(F**, **G**, **H)** Individual AP deviations during standing with 30 Hz vibration plotted against deviations during quiet standing in participants of HY, HE, and FR groups, respectively. **(I**, **J)** Hip medio-lateral (ML) deviations during standing with eyes open (EO), and eyes closed (EC), respectively. **(K**, **L**, **M)** Individual ML deviations during standing with 40 Hz vibration plotted against deviations during quiet standing in participants of HY, HE, and FR groups, respectively. **(N**, **O**, **P)** Individual ML deviations measured during standing with 30 Hz vibration plotted against deviations during quiet standing in participants of HY, HE, and FR groups, respectively. Designations as in Fig 3.

**Fig 6:**
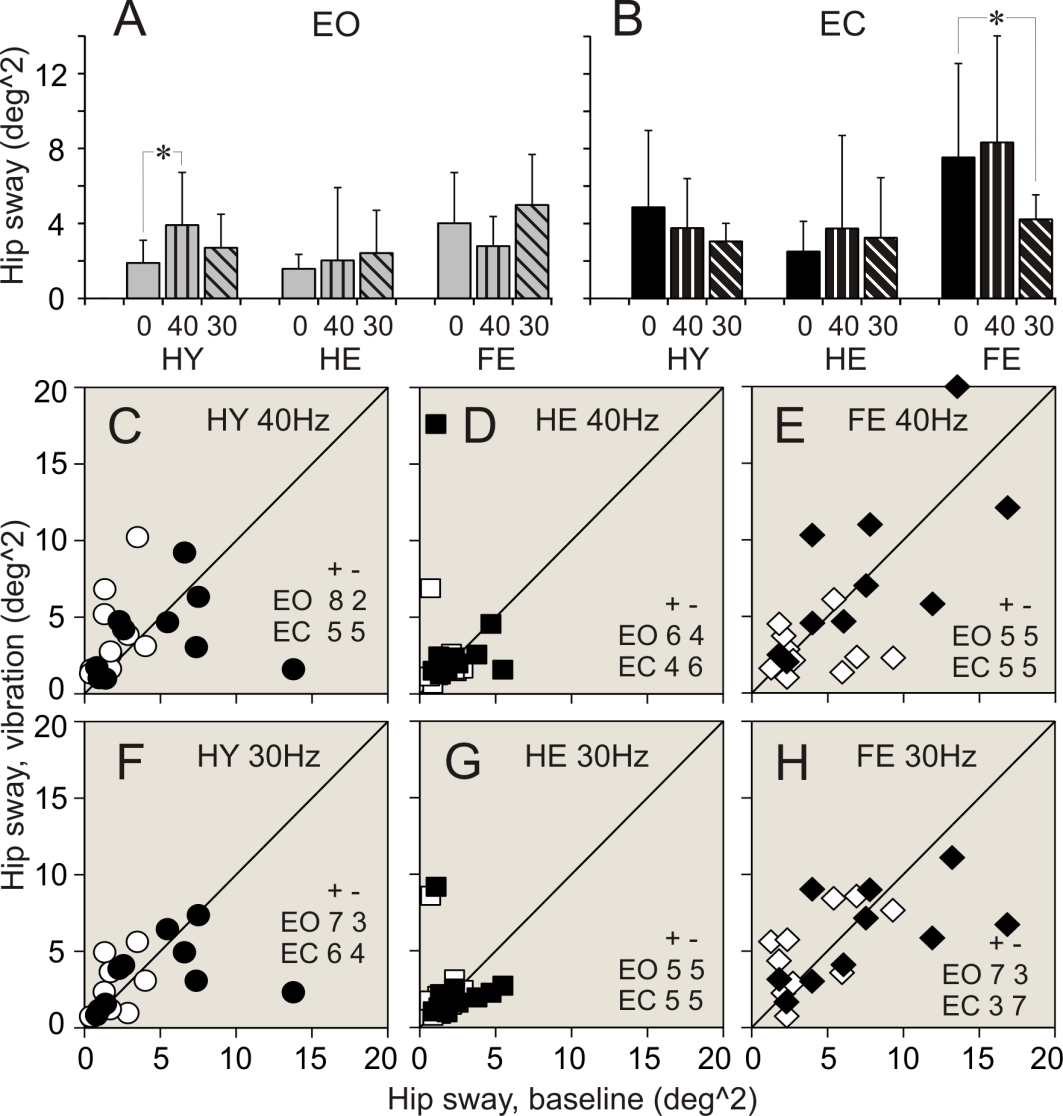
Hip angular sway during standing with and without vibration of ankle muscles. **(A**, **B)** Hip sway during standing with eyes open (EO), and eyes closed (EC), respectively. **(C**, **D**, **E)** Individual hip sway during standing with 40 Hz vibration are plotted against sway during quiet standing in participants of HY, HE, and FR groups, respectively. **(F**, **G**, **H)** Individual hip sway measured during standing with 30 Hz vibration plotted against sway during quiet standing in participants of HY, HE, and FR groups, respectively. Designations as in Fig 3.

**Fig 7:**
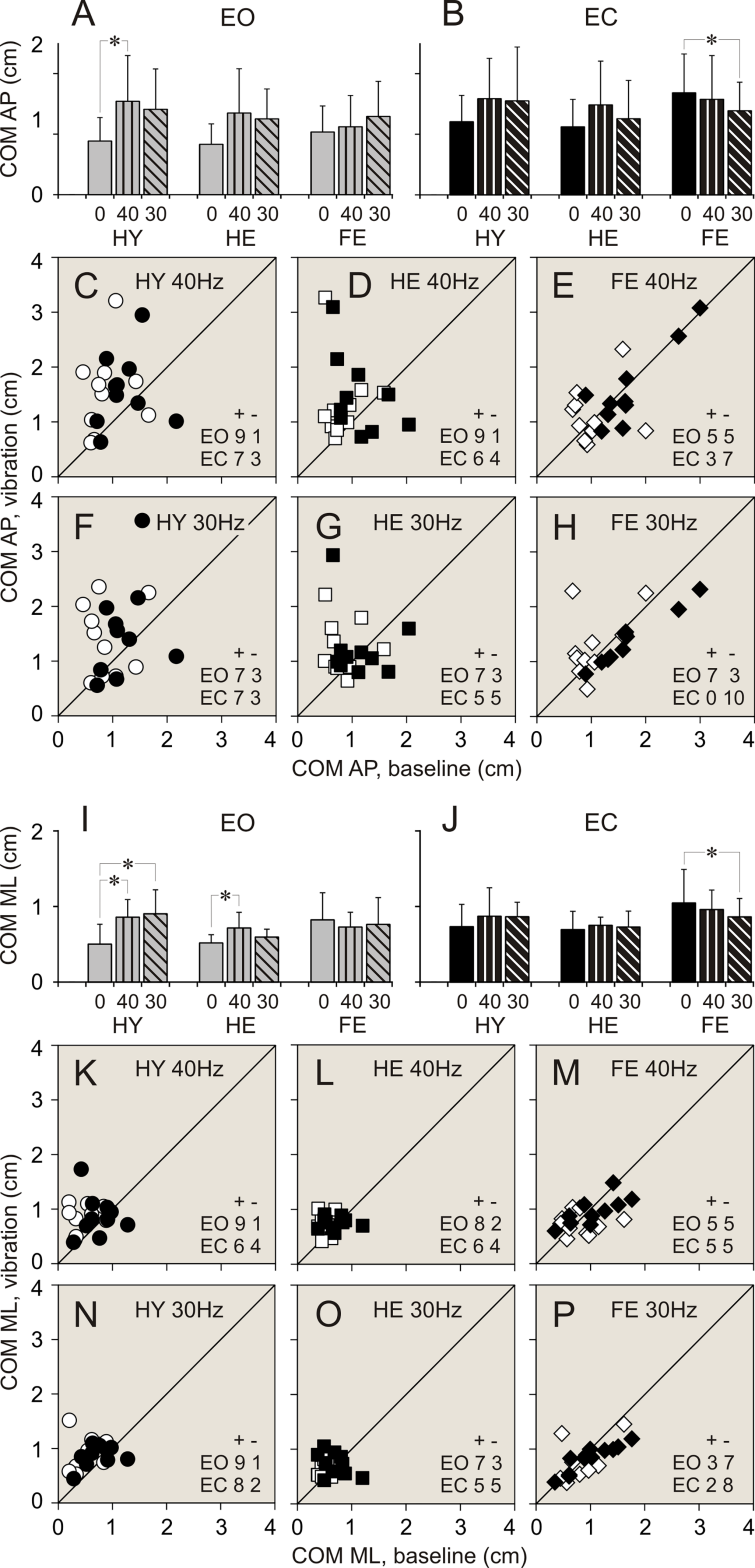
COM linear displacements during standing with and without vibration of ankle muscles. **(A**, **B)** COM anterio-posterior (AP) displacements during standing with eyes open (EO), and eyes closed (EC), respectively. **(C**, **D**, **E)** Individual AP displacements during standing with 40 Hz vibration plotted against displacements during quiet standing in participants of HY, HE, and FR groups, respectively. **(F**, **G**, **H)** Individual AP displacements during standing with 30 Hz vibration plotted against displacements during quiet standing in participants of HY, HE, and FR groups, respectively. **(I**, **J)** COM medio-lateral (ML) displacements during standing with eyes open (EO), and eyes closed (EC), respectively. **(K**, **L**, **M)** Individual ML displacements during standing with 40 Hz vibration plotted against displacements during quiet standing in participants of HY, HE, and FR groups, respectively. **(N**, **O**, **P)** Individual ML displacements during standing with 30 Hz vibration plotted against displacements during quiet standing in participants of HY, HE, and FR groups, respectively. Designations as in Fig 3.

**Fig 8:**
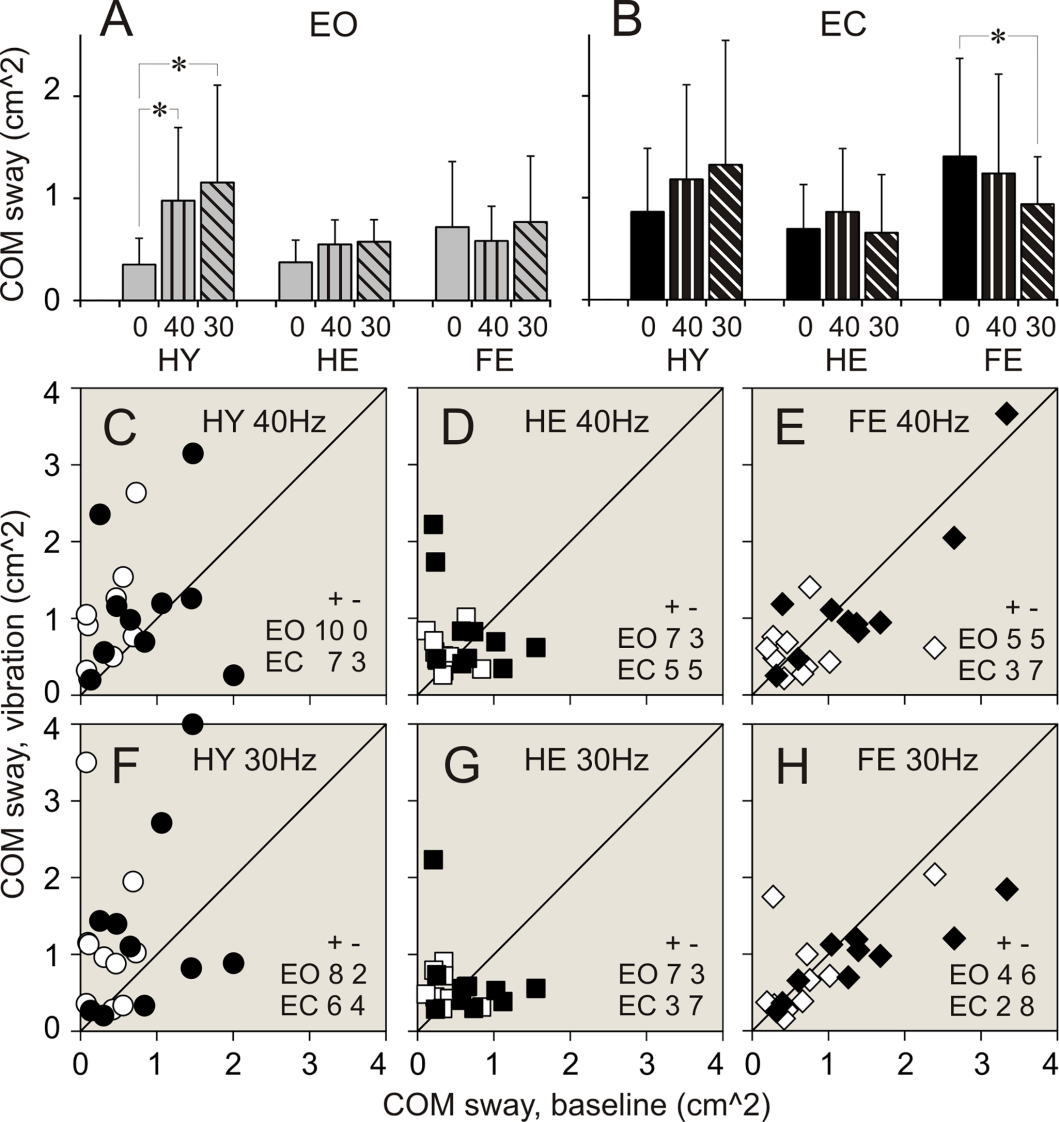
COM linear sway during standing with and without vibration of ankle muscles. **(A**, **B)** COM sway during standing with eyes open (EO), and eyes closed (EC), respectively. **(C**, **D**, **E)** Individual COM sway during standing with 40 Hz vibration plotted against sway during quiet standing in participants of HY, HE, and FR groups, respectively. **(F**, **G**, **H)** Individual COM sway during standing with 30 Hz vibration plotted against sway during quiet standing in participants of HY, HE, and FR groups, respectively. Designations as in Fig 3.

Data of balance parameters during quiet standing and during 40 and 30 Hz vibration of ankle muscles are shown in Tables 2–4 and Figs 3–8. Tables present parameter values during standing in three different conditions, and results of comparison of baseline and vibration parameters in each group. Tables present also numbers of participants of a group, whose balance parameter increased (+) or decreased (-) during standing during ankle vibration, and results of their comparison using χ^2^ test. Figures included bar and scatter plots. Bar plots display average balance parameters for each group of participants. In scatter plots, balance parameters during muscle vibration are plotted against baseline balance parameters during quiet standing for each participant in a group. The numbers of participants of a group, whose parameter during vibration of ankle muscles either increased or decreased, are indicated in scatter plots.

**Table 3:** Hip angular deviations and sway during standing without and with calves’ vibration. Designations as in Table 2.

			Healthy Young	Healthy Elderly	Fall Risk
**Hip AP** **(cm)**	Eyes open	Baseline	2.33±0.89	1.82±0.70	2.91±1.0
		40 Hz	3.70±1.35	3.12±3.88	2.77±0.80
			F(1,9) = 19.40 **p =** **0.0017**	F(1,9) = 1.01 p = 0.3419	F(1,9) = 0.15 p = 0.7035
			*(+) 10 (-) 0* ***p =*** ***0*.*0016***	*(+) 5 (-) 5 p = 1*.*0*	*(+) 4 (-) 6 p = 0*.*5271*
		30 Hz	2.92±1.22	2.62±1.63	3.56±1.24
			F(1,9) = 3.14 p = 0.1100	F(1,9) = 1.94 p = 0.1971	F(1,9) = 4.40 **p = 0.0653**
			*(+) 6 (-) 4 p = 0*.*5271*	*(+) 8 (-) 2* ***p = 0*.*0568***	*(+) 8 (-) 2* ***p = 0*.*0568***
	Eyes closed	Baseline	2.97±1.11	2.34±0.53	4.62±2.28
		40 Hz	3.11±1.31	3.32±3.27	4.36±2.13
			F(1,9) = 0.13 p = 0.7317	F(1,9) = 0.82 p = 0.3886	F(1,9) = 0.37 p = 0.5598
			*(+) 4 (-) 6 p = 0*.*5271*	*(+) 3 (-) 7 p = 0*.*2059*	*(+) 4 (-) 6 p = 0*.*5271*
		30 Hz	3.05±0.96	3.24±3.21	4.23±1.3
			F(1,9) = 0.05 p = 0.8330	F(1,9) = 0.71 p = 0.4219	F(1,9) = 0.60 p = 0.4576
			*(+) 7 (-) 3 p = 0*.*2059*	*(+) 5 (-) 5 p = 1*.*0*	*(+) 4 (-) 6 p = 0*.*5271*
**Hip ML** **(cm)**	Eyes open	Baseline	1.01±0.32	1.18±0.28	1.69±0.62
		40 Hz	1.18±0.41	1.15±0.31	1.32±0.38
			F(1,9) = 1.89 p = 0.2024	F(1,9) = 0.10 p = 0.7546	F(1,9) = 2.35 p = 0.1598
			*(+) 7 (-) 3 p = 0*.*2059*	*(+) 4 (-) 6 p = 0*.*5271*	*(+) 3 (-) 7 p = 0*.*2059*
		30 Hz	1.07±0.32	1.10±1.07	1.73±0.64
			F(1,9) = 0.34 p = 0.5760	F(1,9) = 0.34 p = 0.5746	F(1,9) = 0.02 p = 0.8872
			*(+) 6 (-) 4 p = 0*.*5271*	*(+) 5 (-) 5 p = 1*.*0*	*(+) 4 (-) 6 p = 0*.*5271*
	Eyes closed	Baseline	1.58±0.60	1.40±0.58	2.19±1.01
		40 Hz	1.45±0.54	1.31±0.40	2.15±0.69
			F(1,9) = 0.43 p = 0.5273	F(1,9) = 0.19 p = 0.6738	F(1,9) = 0.03 p = 0.8687
			*(+) 5 (-) 5 p = 1*.*0*	*(+) 4 (-) 6 p = 0*.*5271*	*(+) 5 (-) 5 p = 1*.*0*
		30 Hz	1.34±0.38	1.11±0.15	1.66±0.48
			F(1,9) = 3.33 p = 0.1014	F(1,9) = 3.13 p = 0.1106	F(1,9) = 5.72 **p =** **0.0405**
			*(+) 3 (-) 7 p = 0*.*2059*	*(+) 5 (-) 5 p = 1*.*0*	*(+) 1 (-) 9* ***p =*** ***0*.*0114***
**Hip sway** **(cm^2)**	Eyes open	Baseline	1.90±1.21	1.58±0.78	4.01±2.70
		40 Hz	3.91±2,81	2.03±1.78	2.79±1.58
			F(1,9) = 6.56 **p =** **0.0306**	F(1,9) = 0.48 p = 0.5049	F(1,9) = 1.49 p = 0.2528
			*(+) 8 (-) 2* ***p = 0*.*0568***	*(+) 6 (-) 4 p = 0*.*5271*	*(+) 5 (-) 5 p = 1*.*0*
		30 Hz	2.70±1.79	2.42±2.28	4.99±2.69
			F(1,9) = 2.35 p = 0.1598	F(1,9) = 1.09 p = 0.3244	F(1,9) = 1.71 p = 0.2232
			*(+) 7 (-) 3 p = 0*.*2059*	*(+) 5 (-) 5 p = 1*.*0*	*(+) 7 (-) 3 p = 0*.*2059*
	Eyes closed	Baseline	4.87±4.11	2.51±1.61	7.54±5.01
		40 Hz	3.76±2.64	3.74±4.96	8.34±6.34
			F(1,9) = 0.64 p = 0.4435	F(1,9) = 0.49 p = 0.5004	F(1,9) = 0.27 p = 0.6149
			*(+) 5 (-) 5 p = 1*.*0*	*(+) 4 (-) 6 p = 0*.*5271*	*(+) 5 (-) 5 p = 1*.*0*
		30 Hz	3.05±0.96	3.24±3.21	4.23±1.30
			F(1,9) = 2.19 p = 0.1733	F(1,9) = 0.37 p = 0.5602	F(1,9) = 5.82 **p =** **0.0391**
			*(+) 6 (-) 4 p = 0*.*5271*	*(+) 5 (-) 5 p = 1*.*0*	*(+) 3 (-) 7 p = 0*.*2059*

**Table 4:** COM linear displacements and sway during standing without and with calves’ vibration. Designations as in Table 2.

			**Healthy Young**	**Healthy Elderly**	**Fall Risk**
**COM AP** **(cm)**	Eyes open	Baseline	0.89±0.39	0.83±0.34	1.03±0.43
		40 Hz	1.54±0.75	1.35±0.73	1.12±0.51
			F(1,9) = 7.02 **p =** **0.0265**	F(1,9) = 3.95 **p = 0.0781**	F(1,9) = 0.20 p = 0.6661
			*(+) 9 (-) 1* ***p =*** ***0*.*011***	*(+) 9 (-) 1* ***p =*** ***0*.*0114***	*(+) 5 (-) 5 p = 1*.*0*
		30 Hz	1.41±0.67	1.25±0.49	1.29±0.58
			F(1,9) = 4.63 **p = 0.0601**	F(1,9) = 4.57 **p = 0.0611**	F(1,9) = 2.22 p = 0.1702
			*(+) 7 (-) 3 p = 0*.*2059*	*(+) 7 (-) 3 p = 0*.*2059*	*(+) 7 (-) 3 p = 0*.*2059*
	Eyes closed	Baseline	1.21±1.08	1.12±0.45	1.68±0.64
		40 Hz	1.59±0.66	1.48±0.72	1.58±0.72
			F(1,9) = 2.67 p = 0.1368	F(1,9) = 1.25 p = 0.2915	F(1,9) = 0.97 p = 0.3500
			*(+) 7 (-) 3 p = 0*.*2059*	*(+) 6 (-) 4 p = 0*.*5271*	*(+) 3 (-) 7 p = 0*.*2059*
		30 Hz	1.55±0.89	1.25±0.63	1.39±0.47
			F(1,9) = 1.63 p = 0.2338	F(1,9) = 0.25 p = 0.6268	F(1,9) = 19.43 **p =** **0.0017**
			*(+) 7 (-) 3 p = 0*.*2059*	*(+) 5 (-) 5 p = 1*.*0*	*(+) 0 (-) 10* ***p =*** ***0*.*0016***
**COM ML** **(cm)**	Eyes open	Baseline	0.50±0.26	0.53±0.11	0.82±0.36
		40 Hz	0.86±0.23	0.72±0.21	0.73±0.20
			F(1,9) = 12.78 **p =** **0.0060**	F(1,9) = 6.58 **p =** **0.0304**	F(1,9) = 0.54 p = 0.4785
			*(+) 9 (-) 1* ***p =*** ***0*.*0114***	*(+) 8 (-) 2* ***p = 0*.*0568***	*(+) 5 (-) 5 p = 1*.*0*
		30 Hz	0.90±0.32	0.59±0.10	0.76±0.35
			F(1,9) = 12.18 **p =** **0.0068**	F(1,9) = 2.69 p = 0.1348	F(1,9) = 0.28 p = 0.6078
			*(+) 9 (-) 1* ***p =*** ***0*.*0114***	*(+) 7 (-) 3 p = 0*.*2059*	*(+) 3 (-) 7 p = 0*.*2059*
	Eyes closed	Baseline	1.21±1.08	1.12±0.45	1.68±0.64
		40 Hz	0.87±0.38	0.75±0.11	0.96±0.26
			F(1,9) = 0.79 p = 0.3976	F(1,9) = 0.45 p = 0.5183	F(1,9) = 0.83 p = 0.3862
			*(+) 6 (-) 4 p = 0*.*5271*	*(+) 6 (-) 4 p = 0*.*5271*	*(+) 5 (-) 5 p = 1*.*0*
		30 Hz	0.86±0.19	0.73±0.21	0.86±0.24
			F(1,9) = 2.34 p = 0.1607	F(1,9) = 0.07 p = 0.7941	F(1,9) = 5.31 **p =** **0.0467**
			*(+) 8 (-) 2* ***p = 0*.*0568***	*(+) 5 (-) 5 p = 1*.*0*	*(+) 2 (-) 8* ***p = 0*.*0568***
**COM sway** **(cm^2)**	Eyes open	Baseline	0.35±0.26	0.37±0.22	0.72±0.64
		40 Hz	0.98±0 72	0.55±0.24	0.58±0.34
			F(1,9) = 11.09 **p =** **0.0088**	F(1,9) = 2.7 p = 0.1345	F(1,9) = 0.35 p = 0.5674
			*(+) 10 (-) 0* ***p =*** **0.0016**	*(+) 7 (-) 3 p = 0*.*2059*	*(+) 5 (-) 5 p = 1*.*0*
		30 Hz	1.16±0.96	0.57±0.22	0.77±0.65
			F(1,9) = 5.97 **p =** **0.0372**	F(1,9) = 3.24 p = 0.1051	F(1,9) = 0.09 p = 0.7698
			*(+) 8 (-) 2* ***p = 0*.*0568***	*(+) 7 (-) 3 p = 0*.*2059*	*(+) 4 (-) 6 p = 0*.*5271*
	Eyes closed	Baseline	0.86±0.62	0.69±0.43	1.40±0.96
		40 Hz	1.18±0.63	0.86±0.62	1.24±0.98
			F(1,9) = 0.91 p = 0.3545	F(1,9) = 0.32 p = 0.5851	F(1,9) = 1.27 p = 0.2892
			*(+) 7 (-) 3 p = 0*.*2059*	*(+) 5 (-) 5 p = 1*.*0*	*(+) 3 (-) 7 p = 0*.*2059*
		30 Hz	1.32±1.20	0.66±0.57	0.94±0.47
			F(1,9) = 1.63 p = 0.2340	F(1,9) = 0.02 p = 0.8861	F(1,9) = 6.47 **p =** **0.0316**
			*(+) 6 (-) 4 p = 0*.*5271*	*(+) 3 (-) 7 p = 0*.*2059*	*(+) 2 (-) 8* ***p = 0*.*0568***
			**Healthy Young**	**Healthy Elderly**	**Fall Risk**
**COM AP** **(cm)**	Eyes open	Baseline	0.89±0.39	0.83±0.34	1.03±0.43
		40 Hz	1.54±0.75	1.35±0.73	1.12±0.51
			F(1,9) = 7.02 **p =** **0.0265**	F(1,9) = 3.95 **p = 0.0781**	F(1,9) = 0.20 p = 0.6661
			*(+) 9 (-) 1* ***p =*** ***0*.*011***	*(+) 9 (-) 1* ***p =*** ***0*.*0114***	*(+) 5 (-) 5 p = 1*.*0*
		30 Hz	1.41±0.67	1.25±0.49	1.29±0.58
			F(1,9) = 4.63 **p = 0.0601**	F(1,9) = 4.57 **p = 0.0611**	F(1,9) = 2.22 p = 0.1702
			*(+) 7 (-) 3 p = 0*.*2059*	*(+) 7 (-) 3 p = 0*.*2059*	*(+) 7 (-) 3 p = 0*.*2059*
	Eyes closed	Baseline	1.21±1.08	1.12±0.45	1.68±0.64
		40 Hz	1.59±0.66	1.48±0.72	1.58±0.72
			F(1,9) = 2.67 p = 0.1368	F(1,9) = 1.25 p = 0.2915	F(1,9) = 0.97 p = 0.3500
			*(+) 7 (-) 3 p = 0*.*2059*	*(+) 6 (-) 4 p = 0*.*5271*	*(+) 3 (-) 7 p = 0*.*2059*
		30 Hz	1.55±0.89	1.25±0.63	1.39±0.47
			F(1,9) = 1.63 p = 0.2338	F(1,9) = 0.25 p = 0.6268	F(1,9) = 19.43 **p =** **0.0017**
			*(+) 7 (-) 3 p = 0*.*2059*	*(+) 5 (-) 5 p = 1*.*0*	*(+) 0 (-) 10* ***p =*** ***0*.*0016***
**COM ML** **(cm)**	Eyes open	Baseline	0.50±0.26	0.53±0.11	0.82±0.36
		40 Hz	0.86±0.23	0.72±0.21	0.73±0.20
			F(1,9) = 12.78 **p =** **0.0060**	F(1,9) = 6.58 **p =** **0.0304**	F(1,9) = 0.54 p = 0.4785
			*(+) 9 (-) 1* ***p =*** ***0*.*0114***	*(+) 8 (-) 2* ***p = 0*.*0568***	*(+) 5 (-) 5 p = 1*.*0*
		30 Hz	0.90±0.32	0.59±0.10	0.76±0.35
			F(1,9) = 12.18 **p =** **0.0068**	F(1,9) = 2.69 p = 0.1348	F(1,9) = 0.28 p = 0.6078
			*(+) 9 (-) 1* ***p =*** ***0*.*0114***	*(+) 7 (-) 3 p = 0*.*2059*	*(+) 3 (-) 7 p = 0*.*2059*
	Eyes closed	Baseline	1.21±1.08	1.12±0.45	1.68±0.64
		40 Hz	0.87±0.38	0.75±0.11	0.96±0.26
			F(1,9) = 0.79 p = 0.3976	F(1,9) = 0.45 p = 0.5183	F(1,9) = 0.83 p = 0.3862
			*(+) 6 (-) 4 p = 0*.*5271*	*(+) 6 (-) 4 p = 0*.*5271*	*(+) 5 (-) 5 p = 1*.*0*
		30 Hz	0.86±0.19	0.73±0.21	0.86±0.24
			F(1,9) = 2.34 p = 0.1607	F(1,9) = 0.07 p = 0.7941	F(1,9) = 5.31 **p =** **0.0467**
			*(+) 8 (-) 2* ***p = 0*.*0568***	*(+) 5 (-) 5 p = 1*.*0*	*(+) 2 (-) 8* ***p = 0*.*0568***
**COM sway** **(cm^2)**	Eyes open	Baseline	0.35±0.26	0.37±0.22	0.72±0.64
		40 Hz	0.98±0 72	0.55±0.24	0.58±0.34
			F(1,9) = 11.09 **p =** **0.0088**	F(1,9) = 2.7 p = 0.1345	F(1,9) = 0.35 p = 0.5674
			*(+) 10 (-) 0* ***p =*** **0.0016**	*(+) 7 (-) 3 p = 0*.*2059*	*(+) 5 (-) 5 p = 1*.*0*
		30 Hz	1.16±0.96	0.57±0.22	0.77±0.65
			F(1,9) = 5.97 **p =** **0.0372**	F(1,9) = 3.24 p = 0.1051	F(1,9) = 0.09 p = 0.7698
			*(+) 8 (-) 2* ***p = 0*.*0568***	*(+) 7 (-) 3 p = 0*.*2059*	*(+) 4 (-) 6 p = 0*.*5271*
	Eyes closed	Baseline	0.86±0.62	0.69±0.43	1.40±0.96
		40 Hz	1.18±0.63	0.86±0.62	1.24±0.98
			F(1,9) = 0.91 p = 0.3545	F(1,9) = 0.32 p = 0.5851	F(1,9) = 1.27 p = 0.2892
			*(+) 7 (-) 3 p = 0*.*2059*	*(+) 5 (-) 5 p = 1*.*0*	*(+) 3 (-) 7 p = 0*.*2059*
		30 Hz	1.32±1.20	0.66±0.57	0.94±0.47
			F(1,9) = 1.63 p = 0.2340	F(1,9) = 0.02 p = 0.8861	F(1,9) = 6.47 **p** = **0.0316**
			*(+) 6 (-) 4 p = 0*.*5271*	*(+) 3 (-) 7 p = 0*.*2059*	*(+) 2 (-) 8* ***p = 0*.*0568***

When vibration was applied to ankle muscles during standing with eyes open, the most conspicuous changes in balance parameters were seen in HY group. Few statistically significant changes were found in HE group. There were no statistically significant differences between balance parameters during standing without and with muscle vibration in FR group.

#### 40 Hz vibration

In HY group, an application of 40 Hz vibration to ankle muscles was followed by an expansion of a range of ankle angular deviations and sway in a vast majority of participants (up to 90%). The two-fold increase was seen in all ankle balance parameters (Table 2. Figs 3A, 3C, 3I, 3K, 4A and 4C). A significant increase was seen also in a range of hip AP angular deviations and sway. An increase in these parameters was found in 100% and 90% of participants, respectively (Table 3. Figs 5A, 5C, 6A and 6C). All parameters of COM displacements increased significantly. An expansion of COM linear displacements and sway was seen in 90% and 100% of participants, respectively (Table 4. Figs 7A, 7C, 7I, 7K, 8A and 8C).

In HE group, during 40 Hz vibration of ankle muscles, an increase was found in a range of ankle AP and ML angular deviations. Each of these parameters increased in 80% of participants of the group (Table 3. Fig 2A, 2D, 2I and 2L). In addition, a range of COM ML displacements increased significantly. This increase was seen in 80% of participants (Table 4. Fig 7I and 7L).

In FR group, 40 Hz vibration of ankle muscles did not produced any distinctive changes in balance parameters. Both increases and decreases in a range of all parameters were seen in even numbers of participants of the group (Tables 2–4. Figs 3–8).

#### 30 Hz vibration

In HY group, a statistically significant increase was found in ankle ML angular deviations and sway. An increase in the first of these parameters was seen in 90% of participants, though an increase in the second parameters was seen only in 70% of participants (Table 2. Fig 3I and 3N). In addition, a significant expansion was found in COM ML displacements and sway. These changes were seen 90% and 80% of participants of the group, respectively (Table 4. Figs 7I, 7N, 8A and 8F).

In HE group, an increase in a range, which was just above the level of statistical significance, was found in all ankle angular deviations and sway. Each of these parameters increased in 70–80% of participants (Table 2. Figs 3A, 3F, 3I, 4A and 4G). In addition, a tendency to increase was found in COM AP linear displacements (Table 4. Fig 7A and 7G).

In FR group, a tendency to expand was found in only in hip AP angular deviations (Table 3. Fig 5A and 5H).

### Vibration applied to ankle muscles. Balance parameters during standing with eyes closed

When vibration was applied to ankle muscles during standing with eyes closed, only a few noticeable changes were seen in both groups of healthy participants, HY and HE. In contrast, statistically significant changes in balance parameters occurred in FR group during muscle vibration with the lowest frequency.

#### 40 Hz vibration

In HY group, during an application of 40 Hz vibration to ankle muscles, ankle AP angular deviations increased to a level close to statistical significance (Table 2. Fig 3B and 3C). This increase was seen in 80% of participants of the group. In should be noted that other two ankle balance parameters increases in 80% of participants, but average parameters of the group were statistically insignificant (Table 2). In HE group, a tendency to increase was found in ankle ML angular deviations (Table 2). No distinctive changes were seen in balance parameters in FR group of participants (Tables 2–4).

#### 30 Hz vibration

In HY group, during an application of 30 Hz vibration to ankle muscles, a statistically significant increase was found in ankle ML angular deviations. This increase was seen in 90% of participants of the group (Table 2. Fig 3J and 3N). No other parameters showed any tendencies to increase.

In HE group, 30 Hz vibration did not produce distinct changes in balance parameters. Either increases or decreases in a range of all parameters were seen in even numbers of participants of the group (Tables 2–4).

In FR group, a response to 30 Hz muscle vibration was remarkably different. Ankle AP angular deviations significantly decreased. This decrease was seen in 80% of participants of the group (Table 2. Fig 3B and 3H). In addition, ankle sway showed a tendency to decrease as well (Table 2). Hip ML angular deviations and sway decreased significantly. First of these parameters decreased in 90%, and second parameter decreased in 70% of participants (Table 3. Figs 5J, 5P, 6B and 6H). All COM parameters decreased significantly. COM AP linear displacements decreased in 100% of participants, while both COM ML linear displacements and sway decreased in 80% of participants of the group (Table 4. Figs 7B, 7H, 7J, 7P, 8B and 8H).

## Discussion

In our study we examined postural performance of young healthy adults (HY), healthy elders who did not have balance deficiencies (HE), and older-elders at high risk of falling (FR). Postural performance was assessed using measurements of ankle and hip angular deviations, as well as linear displacements of the COM during standing with eyes open and eyes closed. We analyzed balance parameters in persons when they stood quietly, and when 40 and 30 Hz vibrations were applied bilaterally to ankle muscles, gastrocnemius.

We found that baseline balance parameters assessed in FR persons during quiet standing with eyes open were noticeably different from those in both HY and HE persons. The difference was most conspicuous in hip angular deviations. In FR persons, hip deviations were significantly larger in both AP and ML directions, as compared to healthy persons. This result is consistent with an earlier report that hip sway during standing is significantly larger in frequent fallers in comparison to healthy elderly persons [37]. Another visible difference between FR persons and healthy persons was in body movements in ML direction. ML ankle and hip angular deviations, as well as COM linear displacements were larger in FR persons than those in both HY and HE persons. This result is in line with reports that differences between elderly persons, who did and did not experienced falls, were most pronounced for measures related to the control of lateral stability [38,39].

We did not find any difference between balance parameters assessed during standing with eyes open in both HY and HE persons. This result is opposite to reports that sway of the center of pressure (COP) increases with age [15,40], and various parameters of COP displacements differ significantly between healthy young and healthy elderly persons [41,42,43]. The dissimilarity between results of our experiments and other studies might be related to differences in balance parameters measured and analyzed. It seems more likely, however, that it is a consequence of an accidental selection of participants for our age groups, which were of rather small sample size.

When participants of our study stood quietly with eyes closed, all postural parameters, which we measured, had a tendency to increase in comparison with parameters assessed during standing with eyes open. Even though an increase did not reach statistically significant level in each parameter, a range of all parameters had an unmistakable trend to expand after an exclusion of visual cues. We found that an increase in balance parameters was of similar proportion in all groups of participants. Accordingly, during standing with eyes closed, balance parameters were similar in HY and HE groups of participants, but were larger in the FR group. Obviously, an expansion of a range of balance parameters in FR persons elevated instability of their posture. However, this instability did not reach a severe level, and none of the participants fell during the experimental session.

The impact of the visual system to body equilibrium is well known and described in details in numerous studies (e.g. [44,45,46,47]). Visual perception of surroundings is not an obligatory requirement for maintenance of balance; however, the efficiency of postural performance declines after complete or even partial exclusion of visual cues from spatial orientation. The COP parameters in adults of any age were found to be larger during standing with eyes closed then during standing with eyes open [17,40, 46,48]. In addition, differences between COP parameters during standing in two conditions were shown to increase with age [40,43,49]. Noteworthy, the largest differences were reported in elderly persons who experienced multiple falls [48]. To understand why elimination of visual cues weakens balance during standing we may recall a dual impact of the visual system to control of posture. First, the visual system provides information on position of the body in surroundings. This information is processed in areas of the brain involved in the motor control and, as a result, facilitates or even defines postural adjustments [44,47,50]. Second, illuminated surroundings are perceived by the non-image-forming visual system, which exerts unspecific excitatory influences on various subcortical and cortical areas of the brain, and improves alertness and cognition [51,52]. The efficiency of this system diminishes with age [53].

Mechanical vibration applied to muscle bellies or tendons is an effective tool to elicit muscle contraction [54,55]. An excitation of muscle proprioceptors and tendon organs results in an increase in the firing activity in afferent fibers, particularly in Ia fibers innervating muscle spindles [54,55,56]. Afferent signals elicit postsynaptic excitatory responses in motor neurons of homonymous muscles [57,58]. With an increase in intensity of vibratory stimuli, these postsynaptic responses are potentiated and became efficient to trigger spiking activity of motor neurons [57,58]. The activity of motor neurons evokes compound muscle action potentials in fibers of the parent muscle, which results in contraction of the muscle [59,60].

Vibration of muscles, which are involved in maintenance of posture, results in changes in spatial orientation of the body during standing. For example, vibrations of gastrocnemius or tibialis anterior muscles, which control angular position of the ankle joint, produce backward or forward body sway, respectively [28,49,61,62]. The most effective frequencies of muscle vibration to elicit postural responses or reception of body sway were found to be in the range of 80–100 Hz [28,32]. It was shown that 85 Hz vibration of gastrocnemius muscle produced changes in posture that were similar in both middle-aged and elderly persons [63]. In healthy adults the lowest vibration frequency that produces consistent postural response was estimated as 40 Hz, and threshold frequency for postural response was estimated to be in the range of 25–30 Hz [26]. Whether muscle vibrations with low near-threshold frequencies could produce postural responses in elderly persons at high risk of falling is not clear yet.

In our study we examined how vibration of ankle muscles with low range frequencies affects balance parameters in healthy young persons (HY), healthy elderly persons (HE), and elderly persons at high risk of falling (FR). We found that while standing with eyes open, 40 Hz vibration produced an increase in all but one tested balance parameters in HY persons. In HE persons, 40 Hz vibration increased primarily ankle angular deviations. No statistically significant changes were seen in any balance parameters during such stimulation in FR persons. These observations correspond to results of human and animal studies showing an age-related decline in proprioceptive sensitivity and perception of vibration [64,65].

During vibration with 30 Hz frequency, only ankle angular deviations and COM linear displacements increased in HY persons standing with eyes open. There were no changes in balance parameters in both groups of elderly persons, HE and FR. The data demonstrated that vibration frequencies used in our experiments were well above threshold in HY persons. This corresponds to reports that 20–30 Hz vibrations of tendons of tibialis anterior muscle could elicit postural responses in healthy young adults [31,66]. Our study show that HE persons, who were similar to HY persons in regard to their baseline balance parameters during quiet standing, actually were less responsive to proprioceptive vibratory stimulation than HY persons. This is an indication that the efficiency of neuro-muscular system underlying maintenance of balance declines with age even in persons who are considered healthy. Overall, we found a gradual decay in responsiveness to vibratory proprioceptive stimulation in groups of heathy young, healthy elderly and fall risk older-elderly persons. This observation is consistent with numerous data on age-related decrease in vibrotactile sensitivity [64,67,68,69], a decrease in acuity in perception of joint angular position and movement [68,69,70,71,72,73], and an increase in the latency of postural responses [74,75,76].

We found that in HY and HE persons standing with eyes closed, muscle vibrations with both 40 and 30 Hz frequencies did not produce consistent changes in balance parameters. An absence of distinctive postural responses to vibration in healthy persons during standing with eyes closed corresponds to results of studies showing that exclusion of visual cues elevated threshold for discrimination of vibratory stimuli [64,77,78].

In FR persons, 40 Hz vibration did not consistently changed balance parameters either. However, at variance to healthy persons, in FR persons standing with eyes closed, 30 Hz vibration caused distinctive changes in balance parameters. Remarkably, this vibration was accompanied not by an increase, but on the contrary, by a decrease in ankle and hip angular deviations as well as COM linear displacements. To explain the difference between effects of muscle vibration on balance parameters we can compare functional status of the neuro-muscular system in healthy and FR persons. In young healthy persons, balance parameters are result of properly functioning neuro-muscular system controlling posture. Artificial proprioceptive stimulation can only compromise functioning of the system and worsen maintenance of balance, what actually was found in our experiments. This finding is in a full agreement with reports that muscle vibrations applied during natural joint movements elicited quantitatively erroneous proprioceptive messages concerning both spatial and temporal parameters of movements in healthy persons aged 18–43 years [55]. In elderly persons, functioning of neuro-muscular system controlling posture is not optimal, and became critical in persons vulnerable to falls. Of particular importance are age-related malfunctions in the neural system that affect motor responses to postural perturbations. In aging humans, the number of muscle spindles decreases, and the diameter of surviving spindles reduces [3]. The amount of spinal motor neurons decreases sharply in persons above 60 years of age [79]. Membrane properties of surviving spinal motor neurons alter with age [80,81]. Efficiency of Ia monosynaptic and segmental polysynaptic inputs to motor neurons progressively deteriorate with aging [80,82]. Excitatory postsynaptic responses to activation of cortico-spinal projections decline with age in healthy persons, and dramatically reduce in patients with amyotrophic lateral sclerosis [83]. As a result of these processes, average firing rate of motor units, and their peak discharge rate associated with the latency and force of muscle contraction decrease [84,85,86,87]. Ultimately, age-related deterioration in neural control of antigravitational muscles compromises their ability to withstand the weight of the body during standing and walking, and provokes unintentional fall of a person. Due to these age-related changes in neural signal processing, the motor responses to proprioceptive stimulation became ambiguous. Even small differences in the intensity of proprioceptive stimulation caused distinct responses to muscle vibration in FR persons. Muscle vibration with 40 Hz frequency appeared to be intense enough to generate proprioceptive afferent signals triggering the firing activity in motor units, which resulted in postural responses comparable to responses in healthy persons. At variance, vibratory stimulation with 30 Hz frequency was already insufficient to recruit necessary amount of proprioceptors to produce afferent signals triggering the spiking activity of motor neurons of the parent muscle. Yet, the effect of 30 Hz vibratory stimulation was not negligible. Apparently, this stimulation elicited a near-threshold increase in the excitability of motor neurons. An increased excitability of motor neurons elevated their responsiveness to signals due to natural sway of the body. As a result, this low-frequency vibratory stimulation caused improvements in balance parameters and facilitated maintenance of equilibrium during standing in FR persons.

Results of the study confirmed our hypothesis that low intensity vibration of ankle muscles has diverse effects on balance parameters in persons of different age and health status. The major outcome of the study is a finding that low intensity muscle vibration reduces body sway during standing in elderly persons at high risk of falling. This result is clinically relevant because it suggests that low intensity vibratory stimulation of ankle muscles can improve balance in elderly persons prone to balance malfunctions. We suggest that systematic application of mild vibration to ankle muscles could be beneficial to elderly persons, reducing the risk of falls while standing and walking.

## Supporting information

S1 Table Vibration Study Data Set. Participant’s antropometics, test conditions, balance parameters.
(XLSX)
